# The small GTPases Ras and Rap1 bind to and control TORC2 activity

**DOI:** 10.1038/srep25823

**Published:** 2016-05-13

**Authors:** Ankita Khanna, Pouya Lotfi, Anita J. Chavan, Nieves M. Montaño, Parvin Bolourani, Gerald Weeks, Zhouxin Shen, Steven P. Briggs, Henderikus Pots, Peter J. M. Van Haastert, Arjan Kortholt, Pascale G. Charest

**Affiliations:** 1 Department of Cell Biochemistry, University of Groningen, Groningen, 9747AG, Netherlands; 2 Department of Chemistry and Biochemistry, University of Arizona, Tucson, AZ, 85721-0088, USA; 3 Department of Microbiology and Immunology, Life Sciences Centre, University of British Columbia, Vancouver, British Columbia V6T 1Z3, Canada; 4 Section of Cell and Developmental Biology, Division of Biological Sciences, University of California, San Diego, La Jolla, CA, 92093-0380, USA

## Abstract

Target of Rapamycin Complex 2 (TORC2) has conserved roles in regulating cytoskeleton dynamics and cell migration and has been linked to cancer metastasis. However, little is known about the mechanisms regulating TORC2 activity and function in any system. In *Dictyostelium*, TORC2 functions at the front of migrating cells downstream of the Ras protein RasC, controlling F-actin dynamics and cAMP production. Here, we report the identification of the small GTPase Rap1 as a conserved binding partner of the TORC2 component RIP3/SIN1, and that Rap1 positively regulates the RasC-mediated activation of TORC2 in *Dictyostelium*. Moreover, we show that active RasC binds to the catalytic domain of TOR, suggesting a mechanism of TORC2 activation that is similar to Rheb activation of TOR complex 1. Dual Ras/Rap1 regulation of TORC2 may allow for integration of Ras and Rap1 signaling pathways in directed cell migration.

The migration of cells in response to chemical signals (chemotaxis) is central to normal physiology and is involved in many pathological conditions, such as cancer cell metastasis. The Target of Rapamycin Complex 2 (TORC2) recently emerged as a key, conserved player in chemotaxis^1^. TOR complex 1 (TORC1) and TORC2 are conserved complexes formed by the serine/threonine kinase TOR. TORC1 is a master regulator of cell growth and its biochemistry and regulation is well understood^2^. TORC2 plays evolutionarily conserved roles in controlling F-actin organization, and is involved in the regulation of various processes, including cell survival, protein synthesis, and metabolism^3^,^4^. However, in contrast to TORC1, little is understood as to how TORC2 is regulated in any system. Studies performed in *Dictyostelium*, a widely used model of eukaryotic chemotaxis, suggest that TORC2 acts as an integrator of cell movement and chemoattractant signal relay (when stimulated cells transmit chemoattractants to neighboring cells), thereby promoting group cell migration. In *Dictyostelium*, TORC2 is activated downstream from the chemoattractant (cAMP) receptor cAR1, a Ras guanine exchange factor (GEF)-containing complex termed Sca1C, and the Ras protein RasC, which is required for cAMP production and the signal relay response^5^,^6^,^7^,^8^.

In addition to the RasC-TORC2 pathway, chemoattractant stimulation induces the activation of several other signaling pathways initiated by Ras family GTPases, including RasG and Rap1. RasG is activated, in part, by the Ras GEF RasGEFR, and stimulates phosphatidylinositol 3-kinase (PI3K) activity, thereby controlling the site of F-actin polymerization and directionality of migration^9^,^10^,^11^,^12^. Rap1 is activated downstream of RasG^13^, controls cell-substrate adhesion and promotes cell polarization by regulating F-actin remodeling, through both PI3K and Rac proteins, and by inhibiting myosin assembly at the leading edge, through its effector Phg2^14^,^15^,^16^,^17^,^18^.

Here, using an unbiased proteomics approach to identify novel regulators of TORC2 in chemotaxis, we discovered that Rap1 binds the TORC2 component RIP3/SIN1, and that, in addition to RasC, *Dictyostelium* Rap1 regulates TORC2 activity. Further, we found that this interaction is conserved for human Rap1 and SIN1. Finally, we found that *Dictyostelium* RasC binds the catalytic domain of TOR, which suggests that RasC may regulate TORC2 via a mechanism similar to which Rheb regulates TORC1^19^. We propose that the Ras and Rap1 regulation of TORC2 promotes the integration of Ras and Rap1 signaling pathways in response to chemoattractants, thereby coordinating signal relay with the motility cycle during chemotaxis.

## Results

### Rap1 is a conserved binding partner of TORC2 component RIP3/SIN1 whereas RasC binds TOR

**Figure 1:**
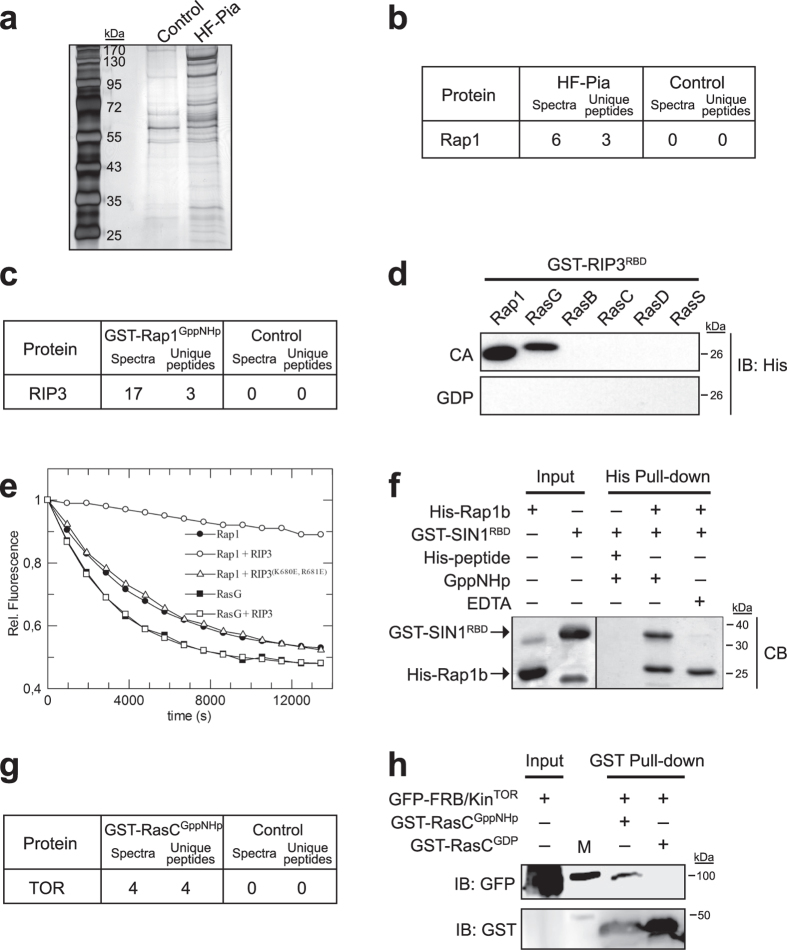
TORC2 binds RasC and Rap1 through different complex components. (**a**) Developed *piaA* null cells expressing HF-Pia were stimulated with the chemoattractant (10 μM cAMP) for 10 sec, followed by sequential His-Flag purification. Proteins pulled-down with HF-Pia were resolved by SDS-PAGE and stained with silver. Wild-type cells were used as control. (**b**) Mass spectrometry data identifying Rap1 co-purifying with HF-Pia. The full list of proteins identified in the HF-Pia pull-down is available in Supplementary Table S1. (**c**) Mass spectrometry data identifying RIP3 in the pull-down performed with GST-Rap1^GppNHp^. (**d**) Interaction between GST-RIP3^RBD^ and His-tagged Rap1, RasG, RasB, RasC, RasD, and RasS was assessed using recombinant, purified proteins *in vitro*, comparing the binding of constitutively active (CA) and GDP-bound (inactive) Rap1/Ras proteins. His-Rap1/Ras proteins were detected by immunoblotting. Immunoblots were cropped, but no other bands were present. Amount of Ras/Rap1 proteins used is shown in Supplementary Fig. S1a. (**e**) Dissociation of mGppNHp from Rap1 and RasG was measured in the presence and absence of 1 μM RIP3^RBD^ or RIP3^(K680E, R681E)RBD^. (**f**) Interaction between GST-SIN1^RBD^ and His-tagged GppNHp-bound or nucleotide free (EDTA) human Rap1b was assessed using recombinant, purified proteins *in vitro*. His-peptide was used as control. Loadings are equivalent between the input and His-Pull-down conditions. Band at ~24 kDa in GST-SIN1^RBD^ input corresponds to free GST. Pull-down proteins were revealed by Commassie Blue (CB) staining. (**g**) Mass spectrometry data identifying TOR in the pull-down performed with GST-RasC^GppNHp^. (**h**) The interaction between GFP-fused TOR catalytic domain (FRB/Kin^TOR^) and GST-fused GppNHp- or GDP-bound RasC was assessed using recombinant, purified proteins *in vitro*. GFP-FRB/Kin^TOR^ and GST-RasC were revealed by immunoblotting. Input represent 33% of protein used in assay. Uncropped gel and immmunoblots are shown in Supplementary Fig. S2. Data are representative of at least two independent experiments.

To identify proteins regulating TORC2 function, we expressed His/Flag-tagged Pianissimo (HF-Pia), the *Dictyostelium* orthologue of mammalian TORC2 essential component Rictor, in *piaA* null cells, and used HF-Pia to purify TORC2 from cells stimulated by the chemoattractant (Fig. 1a). Proteins that co-purify with HF-Pia were identified by mass spectrometry. As expected, known components of TORC2, and of protein synthesis and folding complexes co-purify with HF-Pia (see Supplementary Table S1). Of interest, we found that the small GTPase Rap1 was specifically pulled-down with HF-Pia (Fig. 1b). In addition, in a pull-down screen that we previously performed with recombinant, purified GST-fused Rap1 pre-loaded with non-hydrolyzable GppNHp (active state) or GDP (inactive state)^16^, we found that the TORC2 component RIP3 (orthologue of mammalian SIN1) specifically co-purifies with Rap1^GppNHp^ (Fig. 1c). Together with our finding that Rap1 co-purifies with Pia, this observation suggests that Rap1 interacts with TORC2 by directly binding RIP3.

Similar to most Ras/Rap effectors, RIP3/SIN1 contains a Ras Binding Domain (RBD)^20^,^21^. Despite low amino acid sequence conservation, all RBDs have a typical ubiquitin-like fold that facilitates binding to Ras proteins. The RBD of RIP3 was previously shown to be important for TORC2 function in *Dictyostelium* chemotaxis and to bind the active form of the Ras protein RasG, and not RasC, *in vitro*^22^. However, *in vivo*, RasC, and not RasG, promotes TORC2 activation^5^,^23^. To determine if Rap1 directly interacts with the RBD of RIP3 (RIP3^RBD^), we assessed their binding *in vitro*, compared to the binding of RIP3^RBD^ to other *Dictyostelium* Ras proteins (RasB, RasC, RasD, RasG and RasS). We found that only the active forms of Rap1 and RasG bind RIP3^RBD^ *in vitro* (Fig. 1d). To quantify and compare the binding of RIP3^RBD^ to Rap1 and RasG, we performed a guanine nucleotide dissociation (GDI) assay^18^. Nucleotide dependent binding of an effector to a GTP-bound G protein stabilizes the interaction between the G protein and nucleotide. This stabilization results in inhibition of nucleotide dissociation from the G protein/effector complex. RasG and Rap1 were pre-loaded with fluorescent mGppNHp, and then incubated in the presence of excess unlabeled GppNHp with or without RIP3^RBD^. The exchange of mGppNHP for GppNHp was measured by monitoring fluorescence decay. The resulting observed rate constant (*k*_*obs*_) of fluorescence decay was then determined as a measure of effector binding. In the presence of 1 μM RIP3^RBD^, the *k*_*obs*_ for mGppNHp dissociation from Rap1 is greatly reduced (*k*_*obs*_^*Rap1*^ = 5.0 × 10^−5^ s^−1^ versus *k*_*obs*_^*Rap1+RIP3*^ = 1.0 × 10^−7^ s^−1^), whereas a RIP3 RBD mutant that disrupts Ras/Rap1 binding^22^ (see Supplemental Fig. S1b) has no effect, indicating that RIP3 is binding Rap1 with high affinity, and in a nucleotide dependent way (Fig. 1e). On the other hand, the presence of 1 μM RIP3^RBD^ did not inhibit nucleotide exchange on RasG (*k*_*obs*_^*RasG*^ = 5.9 × 10^−5^ s^−1^ and *k*_*obs*_^*RasG+RIP3*^ = 6.0 × 10^−5^ s^−1^). Consequently, the observation that RIP3 binds with high affinity to Rap1^GppNHp^ suggests RIP3 is a Rap1 effector, whereas the lack of high binding affinity of RIP3 for RasG^GppNHp^ suggests RIP3 is not likely an effector of RasG, which is consistent with previous *in vivo* studies^23^. We then asked if Rap1 binding tRIP3 is conserved in mammals by assessing the interaction of human Rap1b to SIN1^RBD^ using purified proteins *in vitro*. We observed that SIN1^RBD^ binds GppNHp-loaded Rap1b and not nucleotide free Rap1b (Fig. 1f). Therefore, our findings suggest that active Rap1 binds TORC2 through the TORC2-specific subunit RIP3/SIN1, and that this interaction is conserved in mammals.

Although others and we have clear evidence that RasC promotes TORC2 activation in response to chemoattractant stimulation in *Dictyostelium* (present study)^5^,^6^, we find that, unlike Rap1, RasC does not bind RIP3 (Fig. 1d). However, a constitutively active RasC mutant was reported to co-purify with RIP3^6^, suggesting a possible indirect interaction. Interestingly, in a screen for potential RasC-interacting proteins, performed using a protocol similar to that described to identify Rap1-interacting proteins in Fig. 1c, we found that TOR kinase itself specifically co-purifies with RasC^GppNHp^ (Fig. 1g). We verified that RasC binds directly to TOR using recombinant, purified proteins *in vitro*. We observed that active RasC, using either GppNHp-loaded or a constitutively active RasC mutant, binds the catalytic domain of TOR [FKBP-Rapamycin Binding (FRB)/kinase domain] with an average of 9 ± 3.3% efficiency *in vitro*, whereas no binding was observed with inactive, GDP-loaded RasC or a constitutively active Rap1 mutant (Fig. 1h and Supplementary Fig. S3). Thus, together with previous observations, these results suggest that RasC activates TORC2 by directly regulating TOR kinase.

### Rap1 regulates the RasC-mediated activation of TORC2

**Figure 2:**
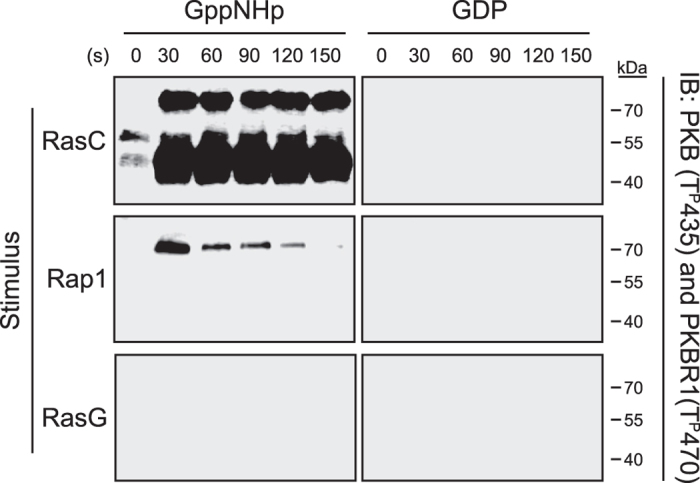
Active RasC and Rap1 both induce TORC2 activation in cell lysates. Cell lysates were stimulated with recombinant, purified, GppNHp- or GDP-bound RasC, Rap1, and RasG for the indicated time. Phosphorylation of PKB (T^P^435; ~50 kDa) and PKBR1 (T^P^470; ~70 kDa) was detected by immunoblotting as a measure of TORC2 activation. Data are representative of at least two independent experiments. Uncropped immunoblots are shown in Supplementary Fig. S5.

To assess whether Rap1, in addition to RasC, can induce TORC2 activation, we first examined the ability of purified, recombinant Rap1, loaded with GppNHp, to induce TORC2 activation in cell lysates. We monitored TORC2 activation by evaluating its phosphorylation of Akt/Protein Kinase B (PKB) and related kinase PKBR1 hydrophobic motif (T^P^435 and T^P^470, respectively)^5^,^7^,^23^. Consistent with previous findings^5^,^6^, RasC^GppNHp^ stimulation of wild-type cell lysates induces the TORC2-mediated phosphorylation of both PKB (~50 kDa band) and PKBR1 (~70 kDa band) (Fig. 2). Interestingly, we found that Rap1^GppNHp^ also stimulates PKBR1 T470 phosphorylation in cell lysates, suggesting activation of TORC2. We do not know why Rap1^GppNHp^ stimulation of cell lysates induces PKBR1 phosphorylation and not that of PKB, but this may suggest an involvement of Rap1 in TORC2 substrate selection and will be the subject of future investigations. Rap1^GppNHp^ and RasC^GppNHp^ do not induce PKBR1 phosphorylation in *piaA* null cell lysates (see Supplementary Fig. S4), confirming that the observed responses are mediated by TORC2. Further, RasG^GppNHp^, RasC^GDP^, and Rap1^GDP^ fail to promote TORC2 activation in wild-type cell lysates, indicating that only active RasC and Rap1, and not RasG, can promote TORC2 activation.

**Figure 3:**
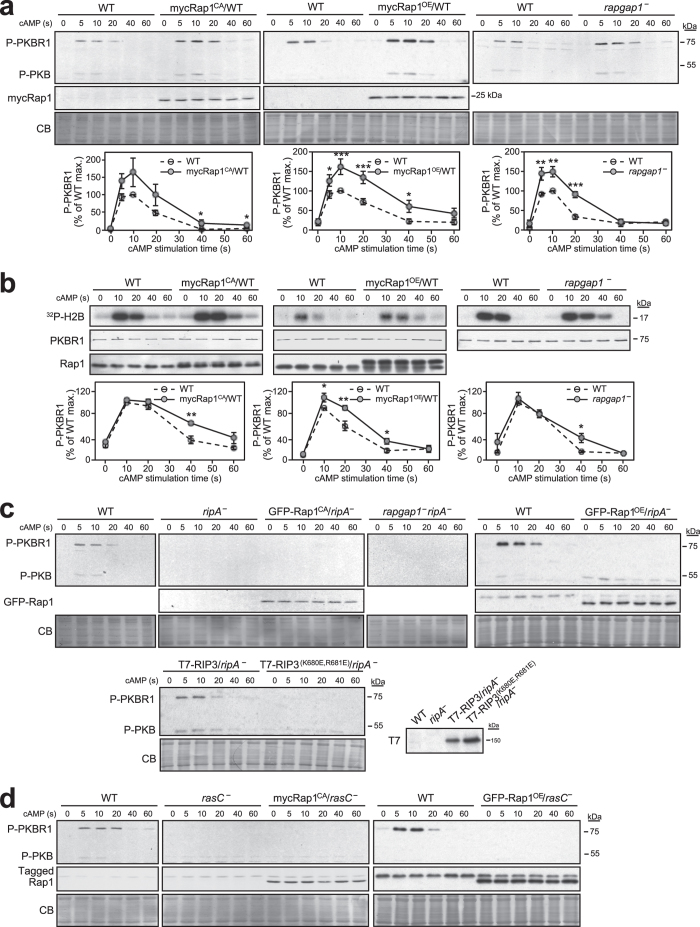
Rap1 positively regulates the RasC-mediated activation of TORC2. (**a**,**c**,**d)** TORC2 phosphorylation of PKB (T^P^435) and PKBR1 (T^P^470) was assessed in wild-type cells (WT), in cells lacking RapGAP1 (*rapgap1*^*−*^), RIP3 (*ripA*^*−*^), both RIP3 and RapGAP1 (*rapgap1*^*−*^*ripA*^*−*^), or RasC (*rasC*^*−*^), and in wild-type, *ripA*^*−*^, and *rasC*^*−*^ cells expressing tagged Rap1^CA^ or Rap1^OE^, and in *ripA*^*−*^ cells expressing either wild-type T7-RIP3 or T7-RIP3^(K680E,681E)^. Cells were stimulated with 10 μM cAMP for the indicated time. PKB/PKBR1 phosphorylation and expression of exogenous Rap1, Rap1^CA^, RIP3, and RIP3^(K680E,681E)^ were revealed by immunoblotting. Equal loading was controlled with Coomassie Blue staining (CB). (**b**) cAMP-induced kinase activity of immunoprecipitated PKBR1 was assessed in the indicated strains using H2B as substrate. H2B phosphorylation was detected by autoradiography and PKBR1 revealed by immunoblotting. Graphs represent mean ± SEM of densitometry quantification data of immunoblots or autoradiographs, from at least three independent experiments, expressed as % of the maximal signal detected in wild-type control cells. *p < 0.05, **p < 0.01, ***p < 0.005. Uncropped immunoblots and autoradiographs are shown in Supplementary Fig. S7.

As cells lacking Rap1 are not viable, to test whether Rap1 controls TORC2 activity *in vivo*, we compared the chemoattractant-induced TORC2 activation in wild-type cells to that in cells displaying elevated Rap1 activity either by overexpressing wild-type Rap1 (Rap1^OE^), expressing a constitutively active Rap1 mutant (Rap1^CA^, G12V mutation), or in cells lacking one of the Rap1-specific GAP, RapGAP1 (*rapgap1* null), in which Rap1 activity is considerably elevated^14^,^24^ (see Supplementary Fig. S6a). In this assay, PKB T435 phosphorylation is sometimes difficult to detect, but PKBR1 T470 phosphorylation is easily traced. As shown in Fig. 3a, we reproducibly observed elevated and extended chemoattractant-induced PKBR1 phosphorylation in all three conditions tested where Rap1 activity is elevated compared to that in wild-type cells. Although the extent of the effect of Rap1^CA^ on PKBR1 phosphorylation varied between experiments, likely due to varying levels of Rap1^CA^ expression related to plasmid copy number per cell as was previously described^25^,^26^,^27^, quantification of the data nonetheless revealed significant (at 40 and 60 sec) or near-significant effects (5 sec, p = 0.053; 10 sec, p = 0.084; 20 sec, p = 0.077). For Rap1^OE^ and *rapgap1* null cells, the effects are strongly significant (for 10 and 20 sec time points, p is between 0.0003 and 0.004). Consistent with an increase in TORC2-mediated PKBR1 phosphorylation, PKBR1 kinase activity is also prolonged in Rap1^CA^, Rap1^OE^, and *rapgap1* null cells, although the effect is not as pronounced as that on PKBR1 T470 phosphorylation (Fig. 3b). This difference is likely due to the fact that the PKB kinase activity is not only regulated by TORC2 but also by other chemotactic effectors and regulatory mechanisms (e.g. GSK3, Protein Phosphatase 2A^28^,^29^).

The increase in PKBR1 phosphorylation observed in *rapgap1* null, and in Rap1^CA^ and Rap1^OE^ cells is not observed in *rapgap1*/*ripA* double null cells or *ripA* null cells expressing Rap1^CA^ or Rap1^OE^, nor is there any TORC2-mediated PKBR1 phosphorylation observed in *ripA* null cells expressing the RIP3^(K680E,R681E)^ mutant (Fig. 3c). These observations indicate that Rap1 interaction with RIP3 is necessary for the observed effect of elevated Rap1 activity on TORC2-mediated PKBR1 phosphorylation and, thus, that Rap1 plays an important role in controlling TORC2 activation. Of note, we sometimes detect constitutive PKB phosphorylation in *ripA* null cells (Fig. 3c and Supplementary Fig. S6b), as was previously observed^7^, but the meaning of which is unknown. Interestingly, however, neither Rap1^OE^ nor Rap1^CA^ induce PKBR1 phosphorylation in cells lacking RasC, suggesting that RasC is essential for TORC2 activation and the Rap1-mediated effect on TORC2 (Fig. 3d). Consequently, we propose that Rap1 positively regulates the RasC-mediated activation of TORC2 in response to chemoattractants.

## Discussion

**Figure 4:**
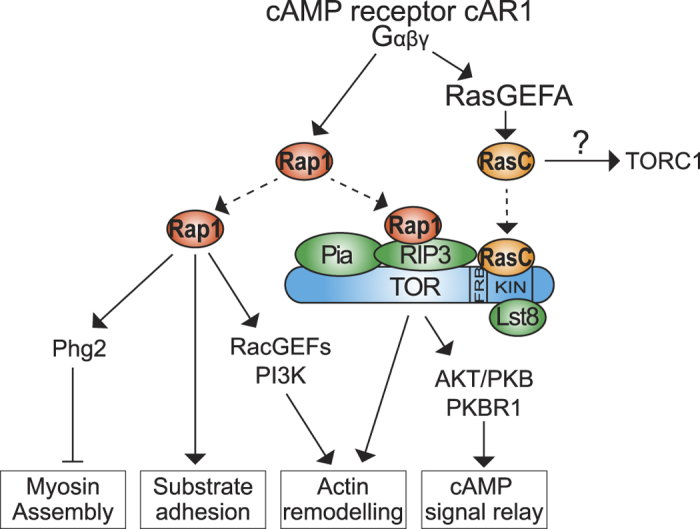
TORC2 is directly regulated by RasC and Rap1 in *Dictyostelium*. In *Dictyostelium*, Chemoattractant (cAMP) stimulation leads to the heterotrimeric G protein (Gαβγ)-dependent activation of RasC (through the RasC specific GEF RasGEFA) and Rap1. In turn, RasC activates TORC2, thereby promoting actin remodeling and the PKB/PKBR1-dependent cAMP production and release (signal relay). Rap1 promotes cell-substrate adhesion and actin remodeling through PI3K and Rac-specific GEFs, and inhibits myosin assembly at the front of migrating cells through Phg2. Here, we show that RasC directly binds the kinase domain of TOR and that Rap1 positively regulates the RasC-mediated activation of TORC2 by directly binding to RIP3/SIN1, providing a possible mechanism through which TORC2 integrates the RasC and Rap1 signals during chemotaxis. We propose that this integration allows linking the signal relay response to cytoskeletal remodeling and substrate adhesion, and, thereby, coordinating group cell migration. Furthermore, our finding that RasC binds TOR suggests RasC may also regulate TORC1. Note: TORC2 components are depicted according to their previously proposed molecular organization^46^.

Our findings reveal new, and likely conserved, mechanisms by which *Dictyostelium* TORC2 is regulated, where both RasC and Rap1 control TORC2 activity through binding of distinct TORC2 components, TOR and RIP3/SIN1, respectively (Fig. 4). In addition, our findings suggest that RasC plays a major role in TORC2 activation and that Rap1 regulates the RasC-mediated activation of TORC2. Whereas we can’t exclude the possibility that Rap1 binds RIP3/SIN1 independently of TORC2, the observation that Rap1 co-purifies with the TORC2 component Pia/Rictor strongly suggests Rap1 binds the TORC2-associated RIP3/SIN1 and, thereby, directly interacts with TORC2. The finding that RasC binds to the catalytic domain of TOR is particularly interesting as it indicates that RasC may activate TORC2 using a mechanism that is similar to the reported Rheb-mediated activation of mTORC1^19^, and that RasC may regulate TORC1 as well. Moreover, as human H-Ras was reported to co-purify with mTORC2^21^, we believe that Ras binding to TOR is likely conserved in mammals. Unfortunately, we were unable to test direct binding of human H-Ras to mTOR *in vitro* due to the difficulty to obtain stable, recombinant mTOR constructs.

Other proteins that we found associated with *Dictyostelium* TORC2 that are known to or could play a role in regulating TORC2 function in chemotaxis include Rac1A and Rab small GTPases, as well as the actin nucleator Formin A. TORC2 interaction with Formin A could represent a potential link between TORC2 and F-actin. In mammals, Rac1 binds TOR and mediates TORC1 and TORC2 localization to specific membranes^30^, and in fission yeast, a Rab-family GTPase was shown to bind and regulate TORC2 signaling^31^. Therefore, our finding that Rac1A and two Rab GTPases, Rab11A and RabC, bind TORC2 suggests that the role of these small GTPases in regulating TORC2 may be conserved in *Dictyostelium*. Finally, although the identification of ribosomal proteins in our HF-Pia pull-down is expected, as HF-Pia was exogenously over-expressed, it is possible that the binding of ribosomal proteins to HF-Pia represents a functional interaction. Indeed, some TORC2 functions were shown to require its association with ribosomes in both yeasts and mammals^32^,^33^.

We propose that the dual RasC- and Rap1-mediated regulation of TORC2 in *Dictyostelium* allows for integration of RasC and Rap1 signaling pathways during chemotaxis and, thereby, coordination of cytoskeletal remodeling, substrate adhesion, and relay of the chemoattractant signal to neighboring cells (Fig. 4). The organized migration of groups of cells is crucial to *Dictyostelium* and human embryonic development as well as to wound healing, and is also involved in cancer metastasis^34^,^35^,^36^,^37^,^38^,^39^. For cells to achieve collective migration as a cohesive group, they must synchronize their movement, which is achieved through cell-cell communication such as signal relay during chemotaxis. Our findings place TORC2 in an ideal position to promote the coordinated regulation of these processes and control group cell migration. Since the interactions between TORC2 and Ras/Rap1 appear conserved, we suggest TORC2 integrates these signals to coordinate cellular migrations in many systems.

## Methods

### Reagents and antibodies

cAMP sodium salt monohydrate was from Sigma-Aldrich (St. Louis, MO, USA) and H2B was from Roche-Genentech (San Francisco, CA, USA). Anti-myc (Myc A7) was from Abcam (Cambridge, MA, USA), anti-Pan Ras (Ab-3; RAS10) was from Calbiochem/EMD Millipore (Billerica, MA, USA), anti-GFP was from Covance (Princeton, NJ, USA), horseradish peroxidase-conjugated secondary antibodies were purchased from Jackson ImmunoResearch Laboratories (West Grove, PA, USA), and DyLight^TM^ secondary antibodies were purchased from Thermo Fisher Scientific (Waltham, MA, USA). Anti-GFP, anti-GST, protein-A sepharose, and GSH affinity resin were from GE Healthcare (Pittsburgh, PA, USA). DdPKBR1 antibody was described previously^40^.

### DNA constructs

His-Flag-Pianissimo (HF-Pia) was generated by adding 6XHis and Flag tags, in tandem, by PCR to the N-terminus of Pia’s coding sequence, which was cloned in the pDM304 vector containing a neomycin resistance cassette. Myc-tagged constitutively active Rap1 (Myc-Rap1^CA^; G12V mutation) obtained from Rick Firtel^14^ was transferred to the pDM358 vector containing a hygromycin resistance cassette. FRB/Kin^TOR^ (aa 1820–2380) was amplified by PCR and subsequently cloned in the pDM317 vector containing N-terminal GFP and neomycin resistance cassette. The RIP3^RBD^ (aa 648–717) used in the *in vitro* binding assay (Fig. 1d), and RasC, were expressed as N-terminal GST-fusion from a pGEX-4T-1 vector. Constitutively active RasC (RasC^CA^, G62L mutation) was generated by the method of Quick change. 6XHis-tagged wild-type and constitutively active forms of the Rap1 and Ras proteins were described previously^41^. The RIP3^RBD^ (aa 511–838) and SIN1^RBD^ (aa 266–374), used in the GDI experiment (Fig. 1e) and *in vitro* binding assay (Fig. 1f), respectively, were expressed as N-terminal GST-fusion from a pGEX-4T-3 plasmid. His-Rap1b was a kind gift of Alfred Wittinghofer.

### Cell culture and strains used

*Dictyostelium* cells were grown in axenic HL5 medium (ForMedium, Hunstanton, Norfolk, UK) at 22 °C and transformants were generated by electroporation. Transformed cells were selected in 20 μg/ml Geneticin or 50 μg/ml Hygromycin B (both from Life Technologies, Grand Island, NY, USA) and expression confirmed by immunoblotting. Aggregation-competent cells were obtained by pulsing cells with 30 nM cAMP every 6 min for 5.5h in 12 mM Na/K phosphate pH 6.1 at 5 × 10^6^ cells/ml. Wild-type cells are AX3 and all transformants and null strains have an AX3 background. *gbpD* null cells were described elsewhere^18^, *piaA* null and *rapgap1* null cells were provided by Peter Devreotes and Rick Firtel, respectively, and were previously described^24^,^42^. The *rapgap1/ripA* double null strain was generated by disrupting *ripA* in the *rapgap1* null background, as described previously^43^.

### Pull-downs and mass spectrometry

Sequential His-Flag purification and identification of the isolated proteins by mass spectrometry was performed as previously described^5^. The pull-down screens for RasC and Rap1 effectors were performed as previously described^16^,^44^.

### *In vitro* binding studies

GST-fused RIP3^RBD^, -SIN1^RBD^, -Rap1, and -RasC were purified by GSH affinity and size exclusion chromatography as previously described^18^,^45^. Purification of His-tagged Rap1 and Ras proteins, and the *in vitro* interaction assay with RIP3^RBD^ was performed as described previously^41^. Purified proteins’ quality was verified on gel and quantified (see Supplemental Fig. S1), and equal amounts were used for each interaction assessed. His-Rap1/Ras proteins were detected by immunoblotting with anti-His monoclonal antibody (sc-8036; Santa Cruz Biotechnology, Dallas, TX, USA). Interaction between His-tagged GppNHp-bound or nucleotide free (EDTA) human Rap1b and GST-SIN1^RBD^ was tested using 25 μg of the purified proteins incubated in binding buffer (50 mM Tris-Cl, 150 mM NaCl, 5 mM MgCl2, 1 mM β-mercaptoethanol, pH 7.5), and proteins were pulled-down with Ni-NTA affinity resin (Qiagen, Valencia, CA, USA) for 2 hours at 4 °C. The beads were washed three times with ice-cold binding buffer containing 500 mM NaCl, and eluted with 300 mM imidazole in binding buffer. The proteins were resolved on SDS-PAGE and revealed by Coomassie Blue staining. GFP-FRB/Kin^TOR^ was isolated from *Dictyostelium* cell lysates using anti-GFP antibody coupled to protein A Sepharose beads. Interaction between GFP-FRB/Kin^TOR^ and GST-fused GppNHp- or GDP-loaded RasC was tested using 25 μg of the purified proteins incubated in binding buffer (50 mM Tris-Cl, 150 mM NaCl, 5 mM MgCl2, 1 mM β-mercaptoethanol, pH 7.5), and proteins were pulled-down with GSH affinity resin. The beads were washed three times with ice-cold binding buffer containing 500 mM NaCl, and eluted with 20 mM glutathione in binding buffer. The proteins were resolved on SDS-PAGE and revealed by GFP and GST immunoblotting.

### Biochemical assays

The guanine nucleotide dissociation inhibition (GDI) assay, Rap1 activity assay, and PKBR1 kinase assay were performed as previously described^14^,^18^,^40^. To test the ability of Rap1, RasC or RasG to activate TORC2 in cell lysates, the GTPases were loaded with GDP or GppNHp as previously described^18^. Aggregation competent wild-type cells were harvested by centrifugation and re-suspended in buffer containing 50 mM Tris-Cl (pH = 7.5), 150 mM NaCl, 5 mM MgCl2, 5 mM DTT, 5% Glycerol. Cells were lysed on 5 μm Nuclepore filter and the lysate was cleared by 16,000 × g centrifugation for 5 min at 4 °C. Total cell lysate protein content and purified Ras/Rap1 were quantified with Bradford reagent and 400 μg of cell lysate was stimulated with 1 μM of nucleotide bound GTPases and samples collected at the indicated times. 50 μg of proteins were loaded on gel for each sample. TORC2 activity was assessed by evaluating the TORC2-mediated phosphorylation of PKB and PKBR1 as described previously^5^, with the exception that an anti-phospho-p70S6K antibody (Cell Signaling Technology, Danvers, MA, USA) was used to detect phosphorylation of PKB/PKBR1’s hydrophobic motif (T^P^435 and T^P^470, respectively). Significance of the data was analyzed using unpaired T-Test.

## Additional Information

**How to cite this article**: Khanna, A. *et al*. The small GTPases Ras and Rap1 bind to and control TORC2 activity. *Sci. Rep.* **6**, 25823; doi: 10.1038/srep25823 (2016).

## Supplementary Material

Supplementary Information
