# Supplementary material for: The small GTPases Ras and Rap1 bind to and control TORC2 activity
Source: Sci Rep. 2016 May 13;6:25823. doi: 10.1038/srep25823 (PMC4865869; doi:10.1038/srep25823)
Supplement: Supplementary Information [file srep25823-s1.pdf]

SUPPLEMENTARY INFORMATION

The small GTPases Ras and Rap1 bind to and control TORC2 activity

Ankita Khanna, Pouya Lotfi, Anita J. Chavan, Nieves M. Montaño, Parvin Bolourani, Gerald Weeks, Zhouxin Shen, Steven P. Briggs, Henderikus Pots, Peter J.M Van Haastert, Arjan Kortholt & Pascale G. Charest.

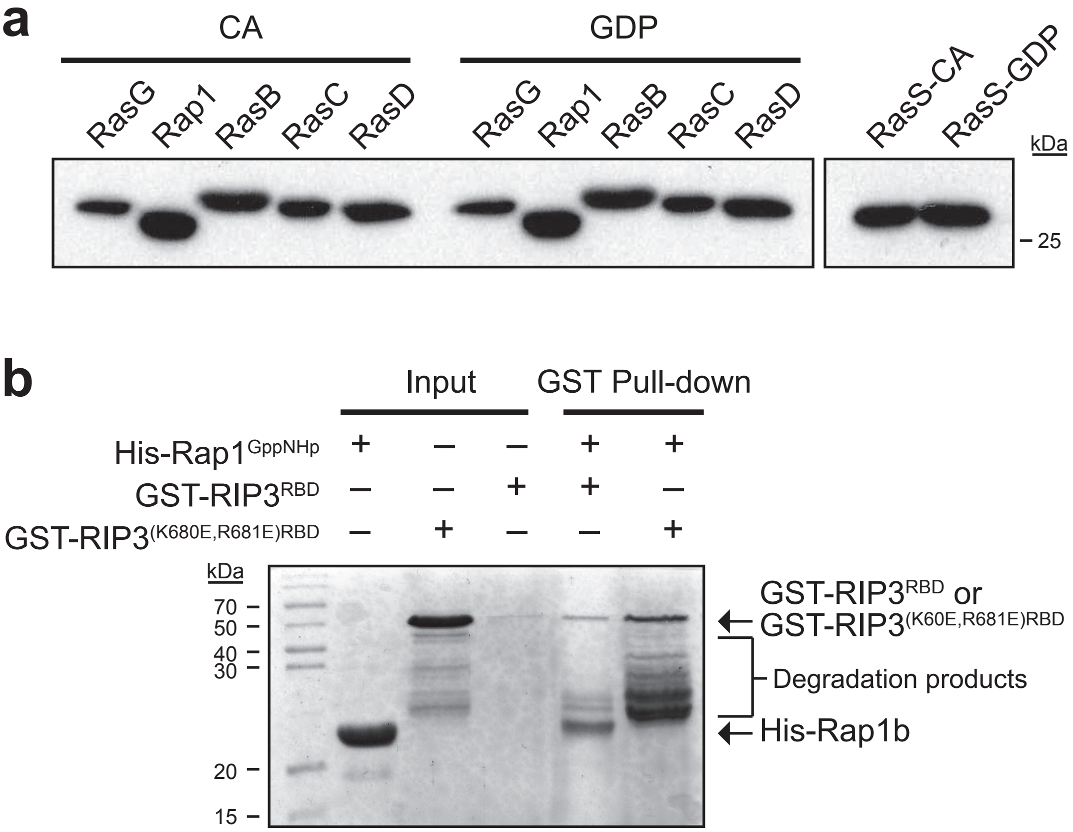

Figure S1. (**a**) Purified constitutively active mutants (CA) or GDP-bound Rap1 and Ras proteins were resolved on SDS-PAGE and revealed by immunobloting to determine their quality and relative quantity. Represents 0.75% of total protein used for the *in vitro* binding assay shown in Fig. 1d. (**b**) Interaction of GST-RIP3^{RBD} and GST-RIP3$^{(K680E,R681E)RBD}$ with his-tagged GppNHp-bound Rap1 was assessed using recombinant, purified proteins *in vitro*. Input corresponds to 20% of the proteins used in the His-Pull-down conditions. Proteins were revealed by Commassie Blue staining. Data are representative of at least two independent experiments.

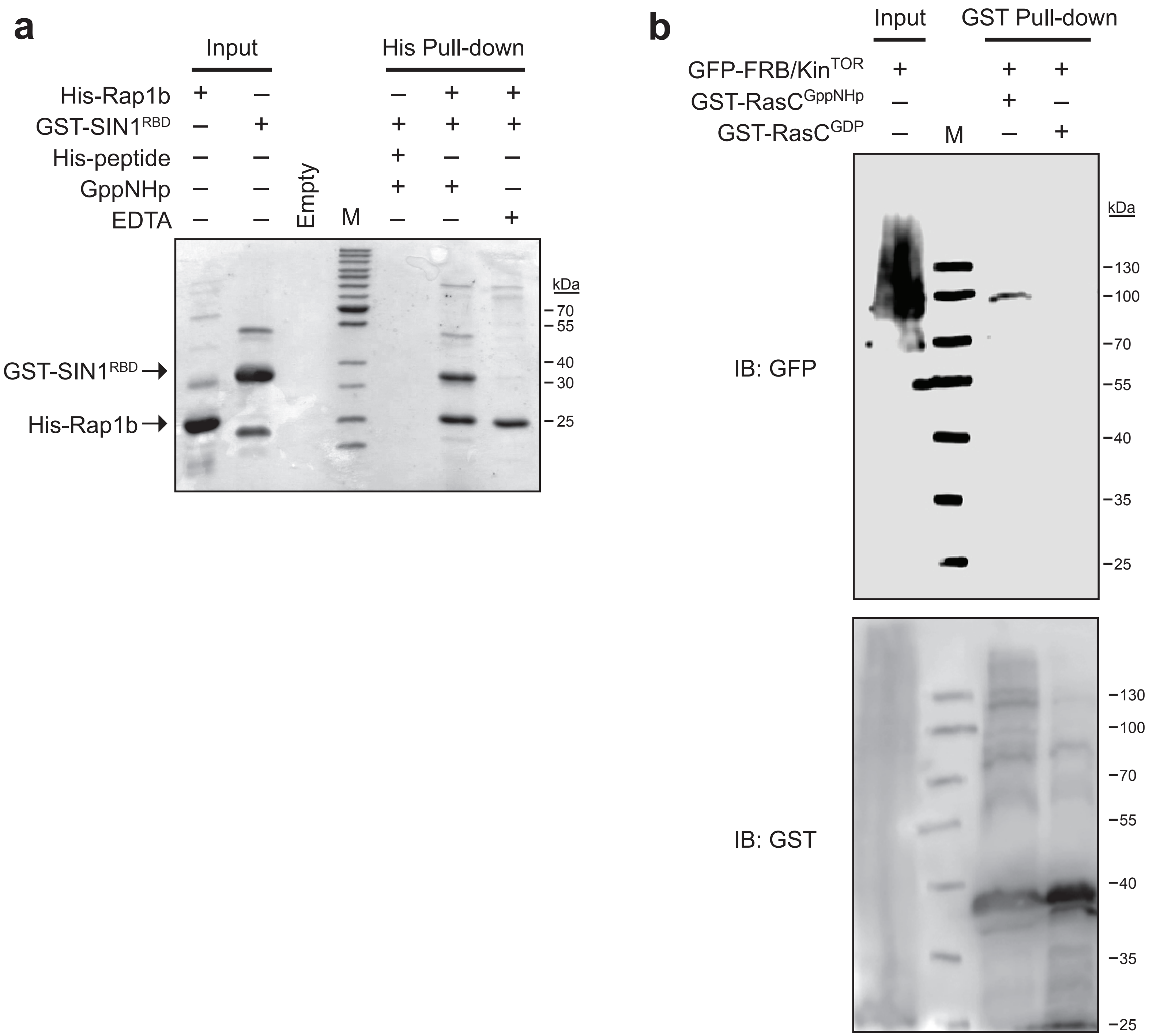

Fig. S2. (**a**) Uncropped stained gel corresponding to data presented in main Fig. 1f. (**b**) Uncropped immunoblots corresponding to data presented in main Fig. 1h.

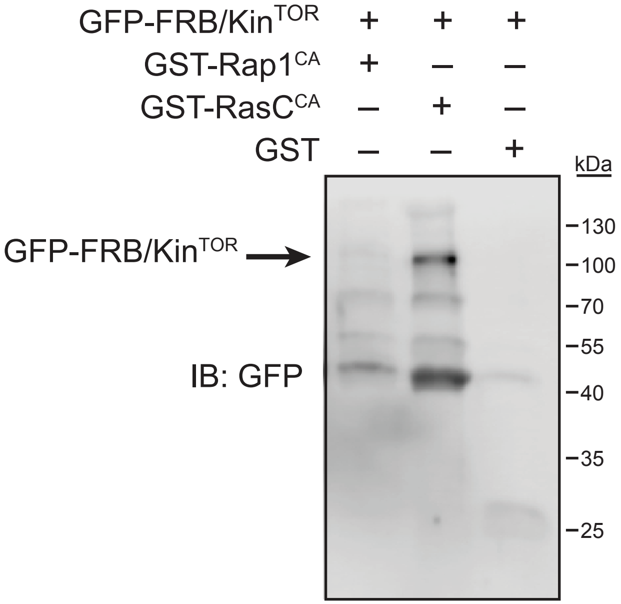

Fig. S3. TOR binds RasC and not Rap1 *in vitro*.
The interaction between GFP-fused TOR catalytic domain (FRB/KinTOR) and GST-fused, constitutively active (CA) Rap1 and RasC was assessed using recombinant, purified proteins in vitro. GFP-FRB/KinTOR was revealed by immunoblotting. GST was used as control.

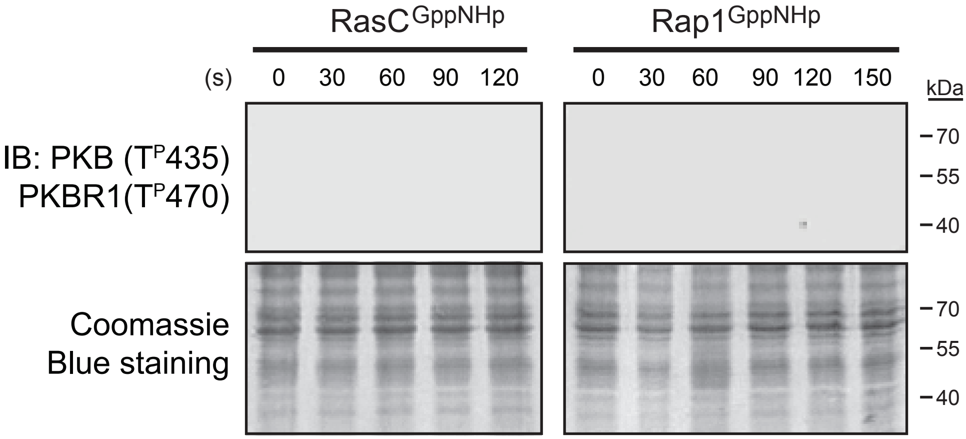

Fig. S4. RasC and Rap1 fail to stimulate PKB and PKBR1 phosphorylation in cells lacking the TORC2 essential component Pia.

piaA- cell lysates were stimulated with recombinant, purified, GppNHp-bound RasC and Rap1 for the indicated time. Phosphorylation of PKBR1 ($T^{P}470$) was detected by immunoblotting as a measure of TORC2 activation. Data are representative of at least two independent experiments.

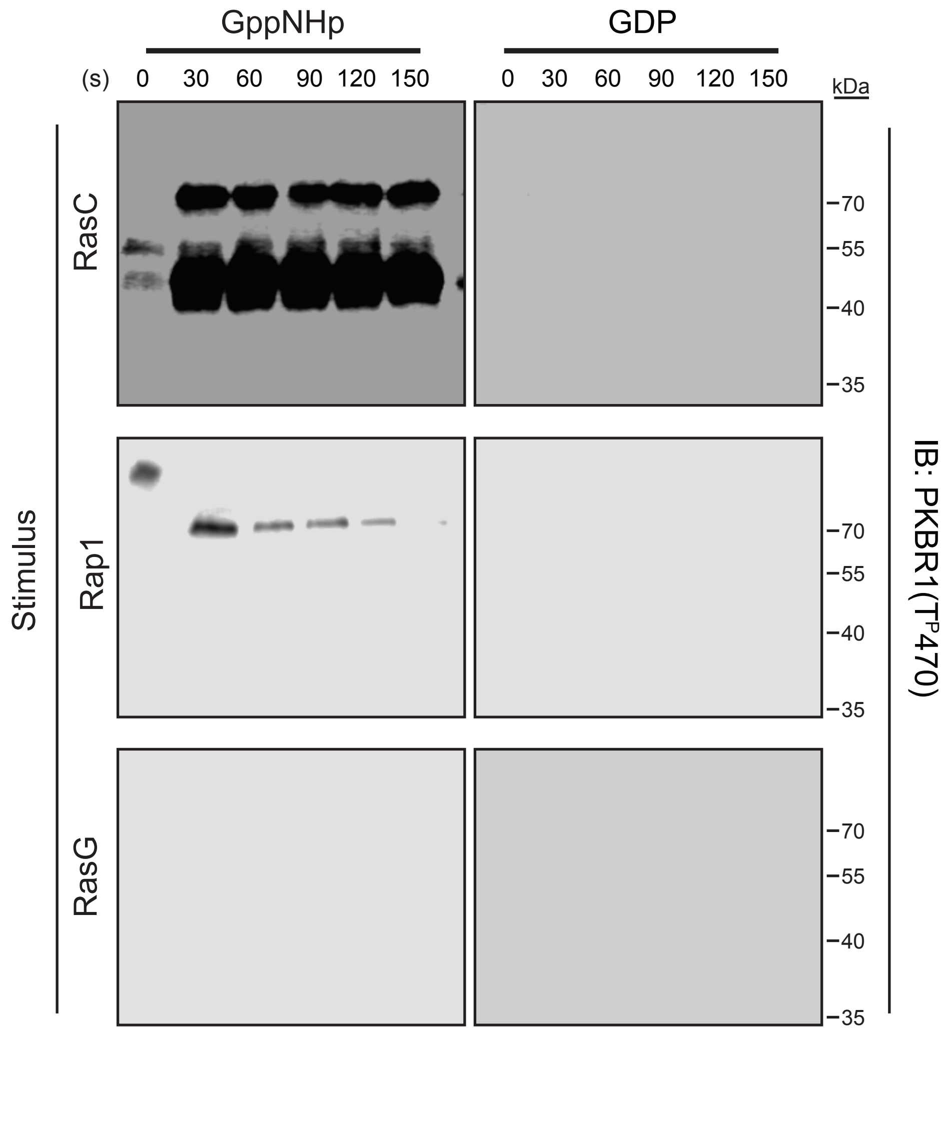

Fig. S5. Uncropped immunoblots corresponding to data presented in main Fig. 2.

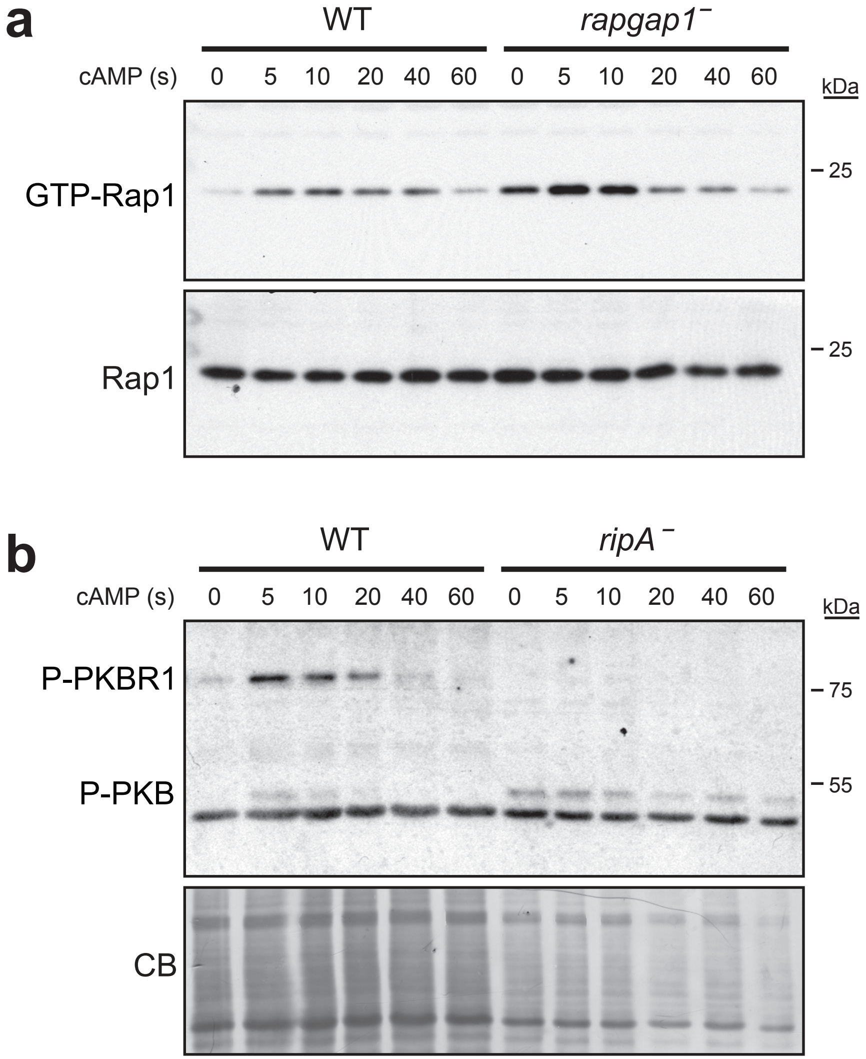

Figure S6. Data supplementary to main Fig. 3. (**a**) Rap1 activity is elevated in cells lacking RapGAP1. Wild-type (WT) and cells lacking RapGAP1 (*rapgap1*$^{-}$) were stimulated for the indicated times with 10 µM cAMP. Active, GTP-bound Rap1 was purified using the Ras binding domain of human RalGDS and both GTP-Rap1 and total Rap1 were revealed by immunoblotting. (**b**) Constitutive PKB phosphorylation is sometimes detected in *ripA*$^{-}$ cells. The meaning of this phosphorylation is unknown.

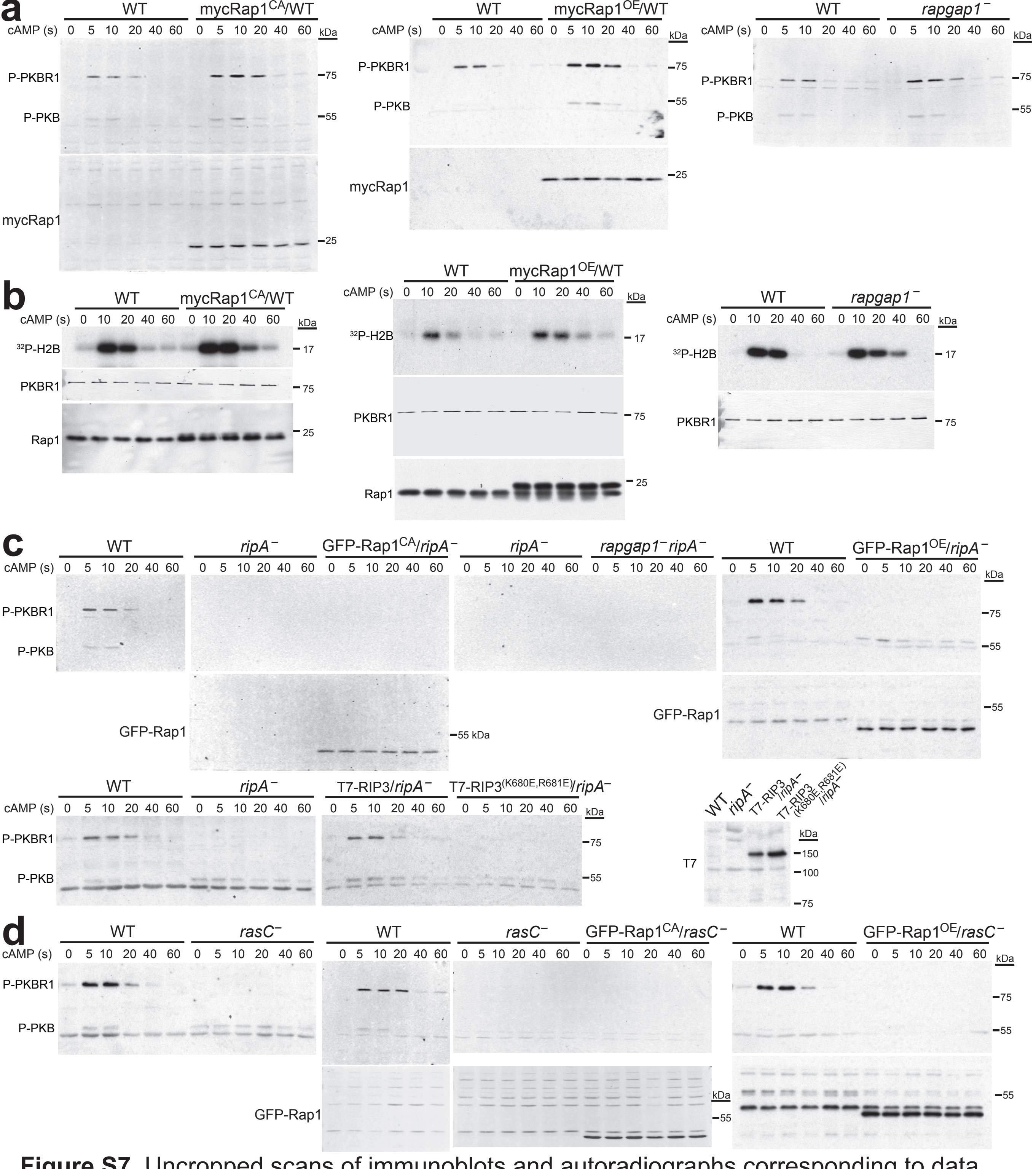

Figure S7. Uncropped scans of immunoblots and autoradiographs corresponding to data in main Fig. 3.

Table S1. Result of the mass spectrometry analysis of the proteins purified with HF-Pia.

Wild-type cells were used as control and proteins that were pulled-down in this control were excluded from the list. Because of the uncertainty, proteins that could be identified only from one peptide were also excluded.

# Spectra	Unique peptides	Protein coverage	Accession number	Gene
**2381**	**50**	**0.47**	**DDB0185055**	**DDB_G0277399 gene: piaA on chromosome: 2 position 8027686 to 8031387**
18	9	0.17	DDB0191163	DDB_G0267400 gene: hspD on chromosome: 1 position 1280990 to 1283391
33	7	0.29	DDB0215391	DDB_G0293742 gene: rps2 on chromosome: 6 position 3277669 to 3278466
29	8	0.44	DDB0229962	DDB_G0279387 gene: rpl21 on chromosome: 3 position 1751733 to 1752215
20	7	0.34	DDB0201667	DDB_G0293000 gene: rps3 on chromosome: 6 position 2556040 to 2556944
15	6	0.30	DDB0230021	DDB_G0277345 gene: rps3a on chromosome: 2 position 7795600 to 7797187
35	5	0.38	DDB0230024	DDB_G0289025 gene: rps7 on chromosome: 5 position 2252902 to 2253483
8	6	0.17	DDB0191262	DDB_G0269146 gene: ifdA on chromosome: 1 position 2640023 to 2641648
13	5	0.32	DDB0230148	DDB_G0290315 gene: rpl23 on chromosome: 5 position 3946402 to 3947228
23	5	0.37	DDB0231057	DDB_G0284093 gene: rps16 on chromosome: 4 position 1558362 to 1559293
10	5	0.13	DDB0233663	DDB_G0276445 gene: DDB_G0276445 on chromosome: 2 position 6984336 to 6986396
22	5	0.23	DDB0231242	DDB_G0278539 gene: rpl5 on chromosome: 3 position 1039561 to 1040650
14	5	0.38	DDB0230153	DDB_G0271298 gene: rpl27 on chromosome: 2 position 140424 to 141268
7	5	0.34	DDB0231058	DDB_G0276415 gene: rps18 on chromosome: 2 position 6891141 to 6891920
11	5	0.13	DDB0305072	DDB_G0281325 gene: DDB_G0281325 on chromosome: 3 position 4191095 to 4192891
9	5	0.37	DDB0230151	DDB_G0280229 gene: rpl24 on chromosome: 3 position 3141943 to 3142654
15	4	0.08	DDB0229857	DDB_G0281605 gene: cfaD on chromosome: 3 position 4701100 to 4702904
9	4	0.06	DDB0304430	DDB_G0284611 gene: ripA on chromosome: 4 position 2346926 to 2349635
9	4	0.30	DDB0230146	DDB_G0288101 gene: rpl22 on chromosome: 5 position 1106395 to 1107007
9	4	0.16	DDB0215016	DDB_G0280037 gene: ddj1 on chromosome: 3 position 3103912 to 3105333
7	4	0.21	DDB0231191	DDB_G0288273 gene: rpl10 on chromosome: 5 position 1312496 to 1313528
5	4	0.11	DDB0231409	DDB_G0291434 gene: pdi2 on chromosome: 6 position 299936 to 301894
6	4	0.13	DDB0231339	DDB_G0277629 gene: rpl7a on chromosome: 2 position 8280728 to 8282193
6	3	0.02	DDB0214908	DDB_G0281569 gene: tor on chromosome: 3 position 4934791 to 4942180
21	3	0.17	DDB0266492	DDB_G0283497 gene: ssr1 on chromosome: 4 position 648667 to 650194
6	3	0.19	DDB0216229	DDB_G0291237 gene: rapA on chromosome: 6 position 98914 to 99707
12	3	0.29	DDB0214936	DDB_G0282379 gene: rpl28 on chromosome: 3 position 6061184 to 6061582
4	3	0.31	DDB0230044	DDB_G0284237 gene: rps12 on chromosome: 4 position 1729814 to 1730224
7	3	0.13	DDB0233351	DDB_G0292698 gene: DDB_G0292698 on chromosome: 6 position 1997307 to 1999014
3	3	0.07	DDB0233917	DDB_G0278053 gene: ilvB on chromosome: 3 position 198473 to 200536
3	3	0.02	DDB0304441	DDB_G0289583 gene: DDB_G0289583 on chromosome: 5 position 2992157 to 2996185
5	3	0.10	DDB0191419	DDB_G0284473 gene: vatC on chromosome: 4 position 2149008 to 2151203
4	3	0.21	DDB0229952	DDB_G0277975 gene: rpl14 on chromosome: 3 position 87206 to 87758
3	3	0.16	DDB0191190	DDB_G0269238 gene: rab11A on chromosome: 1 position 4269122 to 4270264
4	3	0.13	DDB0185073	DDB_G0276441 gene: rpl7 on chromosome: 2 position 6956284 to 6957511
3	3	0.05	DDB0214946	DDB_G0282817 gene: patB on chromosome: 3 position 6309404 to 6312689

3	3	0.06	DDB0215366	DDB_G0293298 gene: mhsp70 on chromosome: 6 position 2761673 to 2763912
5	2	0.25	DDB0169426	DDB_G0276291 gene: DDB_G0276291 on chromosome: 2 position 6564892 to 6565379
4	2	0.14	DDB0230023	DDB_G0280823 gene: rps6 on chromosome: 3 position 1442353 to 1443385
3	2	0.05	DDB0201563	DDB_G0287127 gene: vatA on chromosome: 4 position 5370324 to 5372643
5	2	0.19	DDB0231150	DDB_G0271976 gene: rpl32 on chromosome: 2 position 1095789 to 1096190
2	2	0.05	DDB0191095	DDB_G0289157 gene: cmfB on chromosome: 5 position 2432359 to 2434043
4	2	0.03	DDB0214996	DDB_G0279607 gene: forA on chromosome: 3 position 2388583 to 2393474
5	2	0.17	DDB0231149	DDB_G0279061 gene: rpl31 on chromosome: 3 position 1578225 to 1578923
5	2	0.06	DDB0216376	DDB_G0283279 gene: cdk11 on chromosome: 4 position 446640 to 447716
3	2	0.10	DDB0215012	DDB_G0279411 gene: ctsD on chromosome: 3 position 2104523 to 2105867
4	2	0.05	DDB0231263	DDB_G0281437 gene: lysS on chromosome: 3 position 4498697 to 4500739
2	2	0.02	DDB0237644	DDB_G0268810 gene: DDB_G0268810 on chromosome: 1 position 2146485 to 2149458
3	2	0.09	DDB0185227	DDB_G0273071 gene: vatD-1 on chromosome: 2 position 2787988 to 2789460
9	2	0.14	DDB0231065	DDB_G0277635 gene: rps27 on chromosome: 2 position 8305176 to 8306165
4	2	0.13	DDB0229960	DDB_G0279997 gene: rpl18 on chromosome: 3 position 2837555 to 2838534
2	2	0.03	DDB0232416	DDB_G0277077 gene: DDB_G0277077 on chromosome: 2 position 7466122 to 7468513
3	2	0.05	DDB0191094	DDB_G0291862 gene: rpl3 on chromosome: 6 position 954736 to 956486
2	2	0.05	DDB0201592	DDB_G0294012 gene: atp1 on chromosome: M position 15103 to 16662
4	2	0.17	DDB0219925	DDB_G0283153 gene: cbpD1 on chromosome: 4 position 347658 to 348447
4	2	0.13	DDB0185061	DDB_G0271736 gene: rabC on chromosome: 2 position 718547 to 719569
4	2	0.13	DDB0266464	DDB_G0280313 gene: ssr2 on chromosome: 3 position 3245211 to 3246276
2	2	0.21	DDB0191513	DDB_G0290141 gene: rps17 on chromosome: 5 position 3706333 to 3707026
2	2	0.11	DDB0214822	DDB_G0277869 gene: rac1A on chromosome: 3 position 441220 to 442067
2	2	0.05	DDB0235361	DDB_G0278341 gene: DDB_G0278341 on chromosome: 3 position 722601 to 724035
2	2	0.24	DDB0231061	DDB_G0293700 gene: rps21 on chromosome: 6 position 3013335 to 3014141
2	2	0.24	DDB0231153	DDB_G0270424 gene: rpl35a on chromosome: 1 position 4849685 to 4850344
